# Cultural background and instrument timbre shape emotional and EEG responses to a familiar Middle Eastern melody in Maqam Saba: a crossover clinical trial with implications for music therapy

**DOI:** 10.3389/fnhum.2026.1899966

**Published:** 2026-07-29

**Authors:** Gawaher ElKhatib, Padmakumari Sarada, Sarah Roach, Hanan Saba, Ghizlane Bendriss

**Affiliations:** Premedical Division, Weill Cornell Medicine Qatar, Doha, Qatar

**Keywords:** culture, EEG, Maqam Saba, music intervention, Ney, Oud, PANAS-X, Qanun

## Abstract

Music-based interventions are gaining traction in therapeutic settings, yet evidence remains centered on Western music and populations. This gap is particularly important for Middle Eastern populations, where the Maqam Melodic System—a tonal framework traditionally associated with specific emotions—remains unexplored using modern neuroscientific methods. The Maqam Saba, traditionally associated with sadness, contains quartertones absent from Western scales and unfamiliar to non-Arab listeners, raising the question of whether its emotional associations are universal or culturally specific. This study reports findings from the registered clinical trial NCT04573101, the first to investigate the effects of Arab music on EEG activity at specific sites and emotions using self-reported measures. The full trial examined various variables using a randomized crossover design. The present analysis focuses on responses to “Howa Sahih,” a well-known composition by Zakaria Ahmed, performed on three traditional instruments—Oud, Ney, and Qanun—by professional musicians at Qatar Music Academy. Forty-two participants from diverse ethnic backgrounds were grouped as Arab or non-Arab. Emotions were assessed using PANAS-X and converted to valence, arousal, and dominance (VAD) scores using the NRC VAD Lexicon. EEG activity was recorded at electrode T7, previously linked to emotional processing. Non-parametric tests were used for within- and between-group comparisons. “Howa Sahih” did not consistently evoke negative emotions. Arab listeners showed significantly reduced valence and dominance alongside increased gamma power at T7, a pattern consistent with sadness and emotional processing. Non-Arab listeners instead showed reduced arousal and dominance with increased beta activity, suggesting a rather calming response. These culturally distinct responses were most pronounced with the Ney, the only instrument producing significant between-group differences in both self-reported emotions and gamma activity. The Oud elicited a calming response in non-Arabs, while Qanun increased arousal across the full sample. These exploratory findings suggest that pooling ethnically diverse participants without accounting for these variables may obscure meaningful effects, potentially explaining conflicting findings in music therapy research. Findings describe the possible implications for music intervention design. Maqam Saba on Ney might be promising for eliciting emotional processing in Arab populations. However, further studies are needed to confirm this claim.

## Introduction

Music eliciting negative emotions may have the power to make us feel better. A growing body of evidence suggests that listening to “sad music” can lead to emotional release, consolation, and the regulation of negative mood (Taruffi and Koelsch, 2014). Far from simply reinforcing sadness, this type of music appears to engage introspective processes: it activates the Default Mode Network and promotes self-referential cognition, enabling listeners to process their own emotions (Taruffi et al., 2017). Individuals with higher trait empathy show particularly strong neural responses to sad music, recruiting brain regions involved in compassion and emotional regulation (Taruffi et al., 2021). These findings have contributed to a broader recognition that music-based interventions hold significant promise for clinical and therapeutic settings, from depression and anxiety to neurological disorders such as Parkinson’s disease and dementia (Chou et al., 2026; Aini et al., 2026; Sabbadin et al., 2025; Barnett and Vasiu, 2024).

With advances in neuroscience, the field has evolved from empirical observations of music’s healing properties to neurologically grounded approaches. Thaut et al. (2021) formalized this transition with the development of neurologic music therapy (NMT), a set of standardized clinical techniques based on the neuroscience of music perception and production. NMT and related evidence-based methods have since been applied to speech rehabilitation, motor recovery, and cognitive training, representing a major advance in the therapeutic use of music (Buard et al., 2021).

Yet despite these advances, results across clinical trials remain inconsistent. Meta-analyses of music-based interventions frequently report high heterogeneity and inconclusive evidence, even when the interventions themselves appear well-designed (Sabbadin et al., 2025; Chou et al., 2026). What might explain these conflicting results? Hackett et al. (2021) proposed that music therapy is inherently a precision therapy—one that is deeply connected to and dependent on the individual. They argued that the lack of person-centered methodology, which fails to account for individual differences in clinical, cognitive, and historical attributes, is a key barrier to achieving clarity in the field. We suggest that one such individual attribute that has been largely overlooked is the listener’s cultural background. The overwhelming majority of studies to date have used Western classical music as stimuli and recruited participants from Western populations (Juslin and Laukka, 2003). Musical features traditionally associated with sadness—slow tempo, low pitch, minor mode, and legato articulation (Juslin and Laukka, 2003)—are specific to the Western tonal system. When ethnically diverse participants are pooled together without accounting for their musical enculturation, significant effects may be diluted and masked, producing the very heterogeneity that weakens the evidence base.

This limitation is particularly consequential for the Middle East, where a rich tradition of music and healing dates back over a millennium. As early as the 9th century, scholars such as Al-Farabi (870–950 CE) systematically associated specific melodic modes—known as Maqams—with specific emotional and physiological states (Naroditskaya, 2009; Yöre, 2012). These associations were not merely theoretical: historical accounts describe the use of music in hospitals across the medieval Islamic world, where physicians prescribed specific Maqams for specific ailments, relying on what was essentially passive music listening as a therapeutic modality (Kligman, 2009). Despite this long empirical tradition, the Maqam Melodic System (MMS) remains almost entirely unexplored using modern neuroscientific tools. Unlike Western major and minor scales, the MMS is built on a fixed tonal-spatial component with a free rhythmic-temporal component, and many (but not all) Maqams incorporate quartertones—intervals absent from the Western tonal system—that give them a tonal quality with no Western equivalent (Yaghmour et al., 2021). As a result, Maqams are classified into families of melodic modes defined by their interval signatures rather than by scale names.

Among the most emblematic Maqams is Saba, traditionally and consistently associated with sadness across Arab cultures (Kligman, 2009; Yöre, 2012). The Maqam Saba is characterized by its distinctive interval signature (¾–¾–½) and the presence of quartertones (Yaghmour et al., 2021). But is Saba Maqam truly eliciting sadness? And if so, is it the case only among individuals enculturated in Arab musical traditions, or is it less dependent on culture? These questions matter directly for therapy: if sad music is to be used as a tool for emotional processing, then what constitutes sad music for a given individual must first be established—and this may depend on the listener’s culture.

Several factors beyond the melodic mode itself may shape how a piece of music is associated to specific emotions. Listening to music is a complex cognitive task in which the brain searches for patterns of sounds, recognizes timbres, and retrieves episodic memories associated with them. Expectation—when an episodic memory of the melody is retrieved—and anticipation—after similar elements have been recognized as a pattern—lead the listener’s experience (Harvey, 2020). Given the great diversity of music and cultures, these cognitive processes can produce different responses to the same melody depending on the listener’s cultural background and familiarity with the music. Similarly, the instrument used to play a melody can modulate the emotional response through its timbre—defined as any acoustic property other than pitch, duration, and loudness that allows two sounds to be distinguished. The ability to retain and recognize timbre information is dramatically dependent on musical exposure and therefore on culture.

Three traditional Middle Eastern instruments produce markedly different timbres and were selected for this study. The Oud is a plucked string instrument and the ancestor of the Western lute and guitar; although the two families share structural similarities, their sounds and the emotions they elicit can differ considerably. The Qanun, a 78-string zither also known as the “piano of the East,” is a descendant of the old Egyptian harp and, unlike the traditional piano, can produce microtones. The Ney is an end-blown flute made of giant reed (*Arundo donax*) with no keys or mechanical processing, making it the least processed wind instrument. Its resonances can be adjusted by movements of the lips, tongue, and jaw, and the material is greatly affected by temperature, allowing for the production of microtones and overtones. With this minimal processing, the sound of the Ney is believed in Middle Eastern cultures to invite introspection and is often associated with meditation, prayer, or sadness. We hypothesized that these different instruments would elicit different emotional and EEG responses to the same melody, and that these differences would be modulated by the listener’s cultural background.

Electroencephalography (EEG) provides a non-invasive and temporally precise method for studying cortical responses to music. Recent studies have associated specific EEG frequency bands with emotional processing: gamma band oscillations (25–45 Hz), in particular, have been linked to higher cognitive functions including memory, attention, and emotional processing (Gupta et al., 2023; Xu et al., 2026). Of relevance to this study, increased gamma band power has been observed at left temporal sites during the experience of negative emotions, and specifically during performance on the Saba Maqam at electrode T7 (Yaghmour et al., 2021). Gamma oscillations have also attracted clinical interest as a potential therapeutic target: gamma entrainment through sensory stimulation has been proposed as a novel non-pharmacological approach for Alzheimer’s disease (Manippa et al., 2022), further underscoring the translational relevance of understanding what types of music and listening conditions modulate gamma activity.

The present study reports findings from the registered clinical trial NCT04573101, the first to investigate the effect of Arab music on EEG activity at T7 and self-reported emotions using a combination of EEG and psychometric assessments. The full trial was designed to examine five variables that may influence music perception: Maqam type (Saba and Kurd), instrument timbre (Oud, Ney, and Qanun), pitch (Re and Sol), prior familiarity with the melody, and the presence of quartertones, using a randomized crossover design with 42 participants in Qatar. The present analysis focuses on a specific subset of this trial: the responses to the well-known melody “Howa Sahih El Hawa Ghalab,” a masterpiece in Maqam Saba composed by Zakaria Ahmed and famously interpreted by Umm Kulthum, played on three instruments—Oud, Ney, and Qanun. This melody was selected because it is widely known among Arab listeners, follows the Saba Maqam traditionally associated with sadness, and contains quartertones unfamiliar to non-Arab ears—making it an ideal stimulus to test whether responses to music are culturally universal or culturally specific.

Using a binary approach that combines self-reported emotions (the PANAS-X, converted to valence, arousal, and dominance scores using the NRC VAD Lexicon; Watson and Clark, 1994; Mohammad, 2018) with EEG recorded at electrode T7, we addressed the following three research questions: (1) Does the melody “Howa Sahih” on Maqam Saba elicit emotions with negative valence and increased gamma band activity, regardless of the listener’s cultural background? (2) Does the instrument used to play the melody—Oud, Ney, or Qanun—modulate the EEG activity at T7 and emotional response? (3) Do individuals with Arab and non-Arab backgrounds differ significantly in their EEG activity at T7 and emotional responses to this melody, and if so, for which instrument? Given that this is the first study to investigate culturally mediated responses to a Maqam-based stimulus using EEG, the present analyses are exploratory and intended to generate hypotheses for future confirmatory work.

By characterizing how cultural background and instrument timbre shape emotional and EEG responses to a specific Maqam, this study aims to provide foundational evidence for the development of culturally tailored music interventions in the Middle East. The findings may inform future clinical research investigating whether culturally tailored interventions incorporating Maqam Saba and related musical features could have relevance for conditions such as depression, grief, and post-traumatic stress in Arab populations.

## Methods

### Ethics and registration

The experiment was approved and conducted in accordance with the guidelines of the Weill Cornell Medicine Qatar (WCMQ) Institutional Review Board. The trial was registered on ClinicalTrials.gov under the number NCT04573101. All participants provided written informed consent prior to enrollment.

### Participants

Forty-two participants (26 female, 15 male, 1 other, mean age 38.7 ± 12.9 years, *n* = 39, range 18–69) were recruited via internal email, flyers, and banners at the recruitment site Weill Cornell Medicine Qatar (WCMQ), and through social media. No compensation was given to participants. Participants self-reported their ethnicity during the enrollment process and were accordingly categorized into two groups: Arab (*n* = 21) and non-Arab (*n* = 21). Of these 42 enrolled participants, the number with complete and analyzable data varied across conditions (*N* = 32–34; approximately 16–17 per group), due to missing or unusable data in some recordings. No participant withdrew from the study after recording and no adverse events were recorded; the reduced numbers reflect data completeness rather than dropout. The exact number of participants included in each comparison is reported in Table 1. The Arab group included participants of Sudanese, Jordanian, Palestinian, Saudi, Qatari, Tunisian, Moroccan, Egyptian, and Lebanese ethnic backgrounds. The non-Arab group included participants of Australian, French, Canadian, Indian, American, Italian, Pakistani, British, Kenyan, South African, Filipino, and Bulgarian backgrounds.

**Table 1:** Statistical comparisons of emotion scores (valence, arousal, dominance) and EEG frequency band power (theta, alpha, beta, gamma) at T7 across conditions and groups.

Line number	Comparison	Valence		Arousal		Dominance		Theta		Alpha		Beta		Gamma	
1	*A. Within-subject comparisons—whole sample*														
2	BL vs. NBL	0.232		0.256		0.002** BL > NBL		0.158		0.893		0.006* NBL > BL		0.026* NBL > BL	
3	O7 vs. N7	0.517		0.857		0.234		0.263		0.598		0.440		0.557	
4	Q7 vs. O7	0.686		0.010* Q7 > O7		0.331		0.114		0.601		0.101		0.173	
5	Q7 vs. N7	0.614		0.021* Q7 > N7		0.099		0.817		0.189		0.248		0.411	
6	*B. Within-subject comparisons—by cultural group*														
		Arabs	Non-Arabs	Arabs	Non-Arabs	Arabs	Non-Arabs	Arabs	Non-Arabs	Arabs	Non-Arabs	Arabs	Non-Arabs	Arabs	Non-Arabs
7	BL vs. NBL	0.030*↓	0.394	0.469	0.031* ↓	0.039* ↓	0.020* ↓	0.246	0.407	0.438	0.287	0.084	0.026* ↑	0.043* ↑	0.116
8	BL vs. O7	0.865	0.776	0.796	0.033* ↓	0.234	0.047* ↓	0.758	0.619	0.501	0.653	0.441	0.594	0.433	0.500
9	BL vs. Q7	0.088	0.272	0.438	0.594	0.163	0.730	0.227	0.136	0.836	0.149	0.050* ↑	0.117	0.033* ↑	0.500
10	BL vs. N7	0.030 * ↓	0.125	0.679	0.031* ↓	0.005*↓	0.069 ↓	0.124	0.309	0.179	0.492	0.515	0.959	0.026* ↑	0.500
11	*C. Between-group comparisons (Arab vs. non-Arab)*														
12	BL	0.599		0.175		0.599		0.760		0.634		0.812		0.518	
13	O7	0.809		0.488		0.986		0.708		0.919		0.563		0.029* A > NA	
														***r* = 0.47**	
14	Q7	0.097		0.970		0.142		0.563		0.496		0.683		0.057	
15	N7	0.041* A < NA		0.423		0.049* A < NA		0.946		0.106		0.454		0.038* A > NA	
		***r* = 0.36**				***r* = 0.34**								***r* = 0.41**	

*p*-values are reported from Wilcoxon Signed Ranks Tests (within-subject comparisons) and Mann–Whitney *U*/Kruskal–Wallis Tests (between-group comparisons). Arrows indicate the direction of significant changes. *p ≤ 0.05, **p ≤ 0.01. BL, baseline; NBL, non-baseline (listening to music); O7, Oud; N7, Ney; Q7, Qanun; A, Arab; NA, non-Arab. Effect sizes (*r* = |*Z*|/√*N*) are reported for significant between-group comparisons. *N* varied by condition (valence/dominance on Ney, *N* = 33; gamma, *N* = 34). Bold values correspond to effect sizes for significant differences for between group comparisons.

Inclusion criteria were: (1) being 18 years old or above; (2) living in Qatar; (3) understanding either English or Arabic. Exclusion criteria were: (1) not meeting the inclusion criteria; (2) having auditory impairments; (3) having any known neurological condition (including epilepsy, Alzheimer’s disease, Parkinson’s disease, autism spectrum disorder, etc.); (4) not being able to come to the site (WCMQ); (5) not being able to consent. All enrolled participants had normal audition and no pre-existing neurological conditions.

### Data collection

#### Surveys

Before the EEG recording, participants read and signed the informed consent form and completed a survey collecting information on their age, gender, ethnicity, musical preferences, diet, stress level, music preference, pregnancy status, and whether they played any musical instrument. Surveys were available in both English and Arabic and administered in an online format. In the present analysis, only ethnicity was used to categorize participants into the Arab and non-Arab groups as described above.

#### Self-reported emotions

The Positive and Negative Affect Schedule—Expanded Form (PANAS-X; Watson and Clark, 1994) was used to record participants’ emotions prior to and after the experiment. The PANAS-X is a 60-item self-report tool that measures two higher-order dimensions—positive affect and negative affect—as well as 11 specific affective states, and has demonstrated solid reliability and validity (Watson and Clark, 1994). Written permission to utilize the tool was obtained from its creators. Participants rated each emotion listed on the PANAS-X from 1 (very slightly or not at all) to 5 (extremely). The tool was administered in English for all participants, except for one participant who used the Arabic translation. During the intervention, the tool was modified slightly: rather than rating all emotions, participants were asked to select the single emotion that best described their feeling after listening to each audio clip.

To determine baseline emotion from the pre-experiment PANAS-X, only emotions rated as 5 (extremely) were retained; if no emotion was rated 5, emotions rated 4 (quite a bit) were used instead. Each selected emotion was then assigned quantitative scores for valence, arousal, and dominance using the NRC Valence, Arousal, and Dominance (VAD) Lexicon (Mohammad, 2018), a database containing validated human ratings of valence, arousal, and dominance for more than 20,000 English words. Valence reflects the pleasantness of an emotion, ranging from negative (sad) to positive (joyful); arousal determines the intensity of the emotion; and dominance refers to the degree of control one has over the emotion (Mohammad, 2018). The mean of these scores constituted the baseline emotion (BL). Emotions reported during and after the intervention were processed identically.

#### EEG recording

Skin was prepared using NuPrep abrasive gel and alcohol pads. Using two channels, five shielded electrodes were placed on each participant’s scalp following the International 10–20 system: channel 1 comprised Fp1 (reference), Fp2 (ground), and Oz (recording); channel 2 comprised Cz (reference), Fp2 (ground), and T3 (recording) (Hurless et al., 2013). The electrode labelled T3 in the International 10–20 system corresponds to T7 in the modified combinatorial nomenclature; for consistency with current conventions, we refer to this site as T7 throughout the manuscript. Recording was limited to this single, pre-specified electrode (T7), selected *a priori* on the basis of prior work associating this left-temporal site with gamma activity during Saba-Maqam performance (Yaghmour et al., 2021); the present findings are therefore site-specific. Participants were asked to close their eyes and remain still while listening to the music. Musical stimuli were presented in randomized order using SuperLab 5 stimulus presentation software (Cedrus) from a separate computer, delivered through noise-cancelling headphones (Bose).

EEG signals were recorded using the PowerLab 26T hardware and LabChart 7 software (ADInstruments). Input from the electrodes was amplified using the BioAmp (ADInstruments) before reaching the PowerLab acquisition system. The system recorded at a sampling rate of 128 Hz, for a bandwidth of 0.16–43 Hz. Digital notch filters at 50 and 60 Hz were applied, as well as a built-in digital fifth-order Sinc filter.

Baseline recordings (BL) were obtained immediately before each of the three “Howa Sahih” stimuli analyzed here (O7, N7, Q7), with the participant’s eyes closed in a relaxed state; each stimulus thus had its own immediately preceding baseline. Non-baseline recordings (NBL) were obtained during each stimulus with eyes closed. Only data recorded at T7, a site previously linked to emotional processing and gamma activity during Saba Maqam performance (Yaghmour et al., 2021), are analyzed in the present manuscript.

#### Stimuli

Auditory stimuli were recorded at Qatar Music Academy (Doha, Qatar) by professional musicians. The complete trial design included 24 audio clips of 1 min each, presented in randomized order to address several research questions. The present study analyzes the effect of three of these clips: a familiar melody on Maqam Saba, “Howa Sahih El Hawa Ghalab”—a masterpiece composed by Zakaria Ahmed and famously interpreted by Umm Kulthum—played on pitch Re (D) by three Middle Eastern instruments: Oud (O7), Ney (N7), and Qanun (Q7). The Oud recording was performed by Abdalla Mhoul, the Ney recording by Yassine Ayari, and the Qanun recording by Taoufiq Mirkhan. Audio files are available as Supplementary material.

#### Procedure

Participants were comfortably seated in a chair during the entire session, which did not exceed 1 h including preparation and consent. After signing the consent form and completing the survey described above, participants were given instructions on how to complete the PANAS-X tool before and during the experiment. Electrodes were placed and audio clips were sent randomly one by one. The participant pressed a push button as soon as they heard the music begin, marking the start of the recording of interest. After 1 min, time was given for the participant to select the emotion that best described their feeling, recorded via SuperLab 5. At the end of the session, a final PANAS-X was completed to rate the overall post-experiment emotional state.

### Data processing and analysis

#### EEG data processing

In LabChart 7, markers were used to select the segments of each EEG recording corresponding to each participant’s listening to the three stimuli: O7, N7, and Q7. Baseline (BL) was defined as the period immediately before each audio stimulus, with the participant’s eyes closed in a relaxed state. Using band-pass filtering and fast Fourier transform (FFT), the power spectra of four frequency bands were extracted: theta (4–8 Hz), alpha (8–12 Hz), beta (12–16 Hz), and gamma (25–45 Hz). Mean power was calculated across each frequency range and across all timestamps within each one-minute segment.

### Statistical analysis

All statistical analyses were performed using IBM SPSS version 26.0. Descriptive statistics including the mean power of each frequency band were computed with and without categorization by cultural background, at baseline (BL), during listening to music regardless of instrument (NBL), and during listening to each instrument separately: Oud (O7), Ney (N7), and Qanun (Q7).

Given the small sample size (*N* = 32–34 depending on the condition) and the non-normal distribution of the data, non-parametric tests were used throughout. The Wilcoxon Signed Ranks Test was used for all within-subject paired comparisons, including baseline versus intervention conditions (BL vs. NBL, BL vs. O7, BL vs. N7, BL vs. Q7) and between-instrument comparisons (O7 vs. N7, O7 vs. Q7, N7 vs. Q7). The Mann–Whitney *U* test was used for between-group comparisons of EEG variables (Arab vs. non-Arab), and the Kruskal–Wallis test—mathematically equivalent to the Mann–Whitney *U* test with two groups—was used for between-group comparisons of emotion variables (valence, arousal, and dominance). Outcome variables were the power of each frequency band, and the valence, arousal, and dominance scores derived from the PANAS-X. Independent variables were the presence or absence of the auditory stimulus (BL vs. NBL), the type of instrument (O7, N7, or Q7), and the cultural background group (Arab or non-Arab). Statistical significance was set at *p* ≤ 0.05. Given the exploratory, hypothesis-generating nature of this study and the small number of *a priori* comparisons, *p*-values are reported uncorrected for multiple comparisons; effect sizes are provided to convey the magnitude of effects independently of significance thresholds. The results should therefore be interpreted as exploratory.

## Results

### Listening to the melody “Howa Sahih” was associated with changes in emotions and brainwaves as compared to baseline emotions

**Figure 1:**
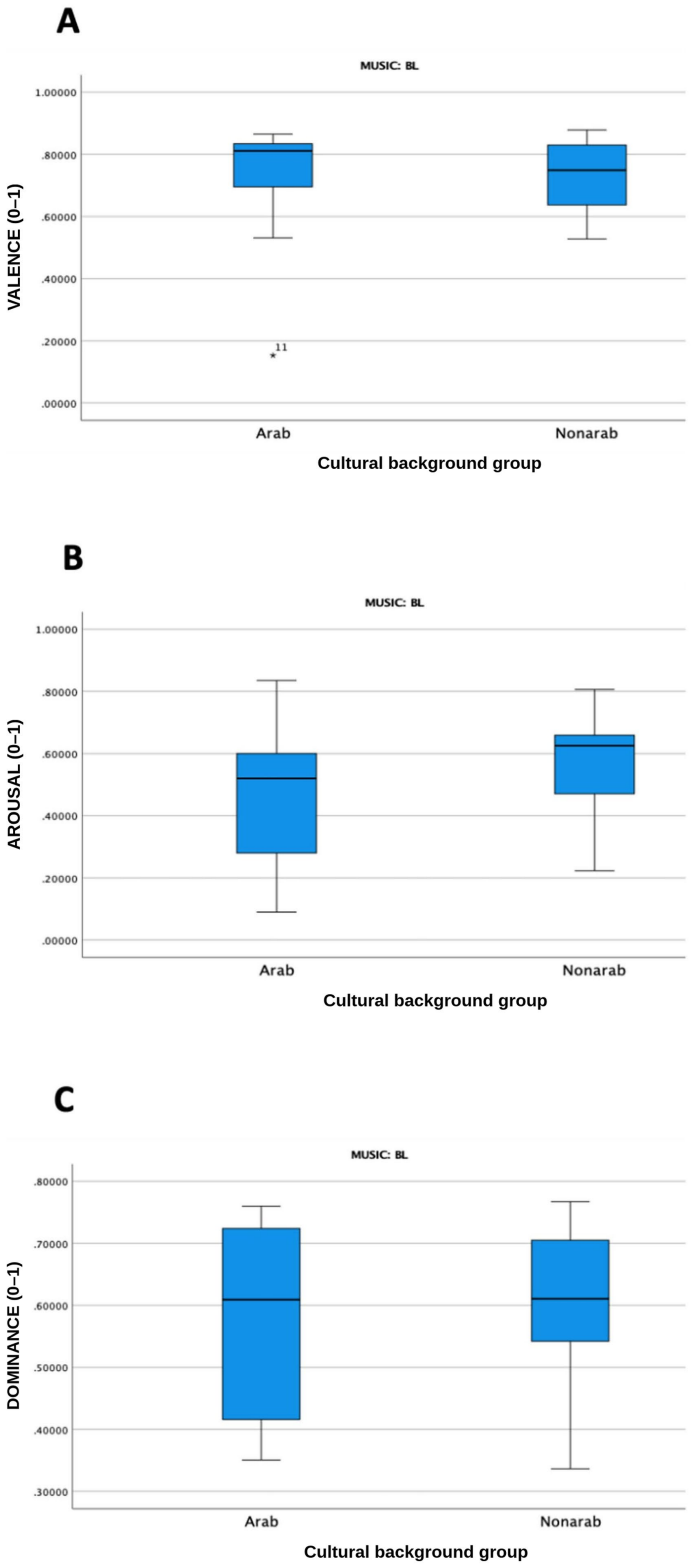
Baseline emotion scores for valence **(A)**, arousal **(B)**, and dominance **(C)** in Arab and non-Arab groups. Boxplots comparing valence, arousal, and dominance scores prior to listening to music. No significant differences were observed between groups (*p* = 0.599, *p* = 0.175, *p* = 0.599, respectively).

All analyses below are exploratory. They concern the three “Howa Sahih” clips (Oud, Ney, Qanun). We first compared baseline emotions (BL, recorded before each clip) between the two groups. We did not find any significant difference between baseline scores of valence, arousal and dominance between Arabs and non-Arabs (Figure 1, Table 1, line 12).

**Figure 2:**
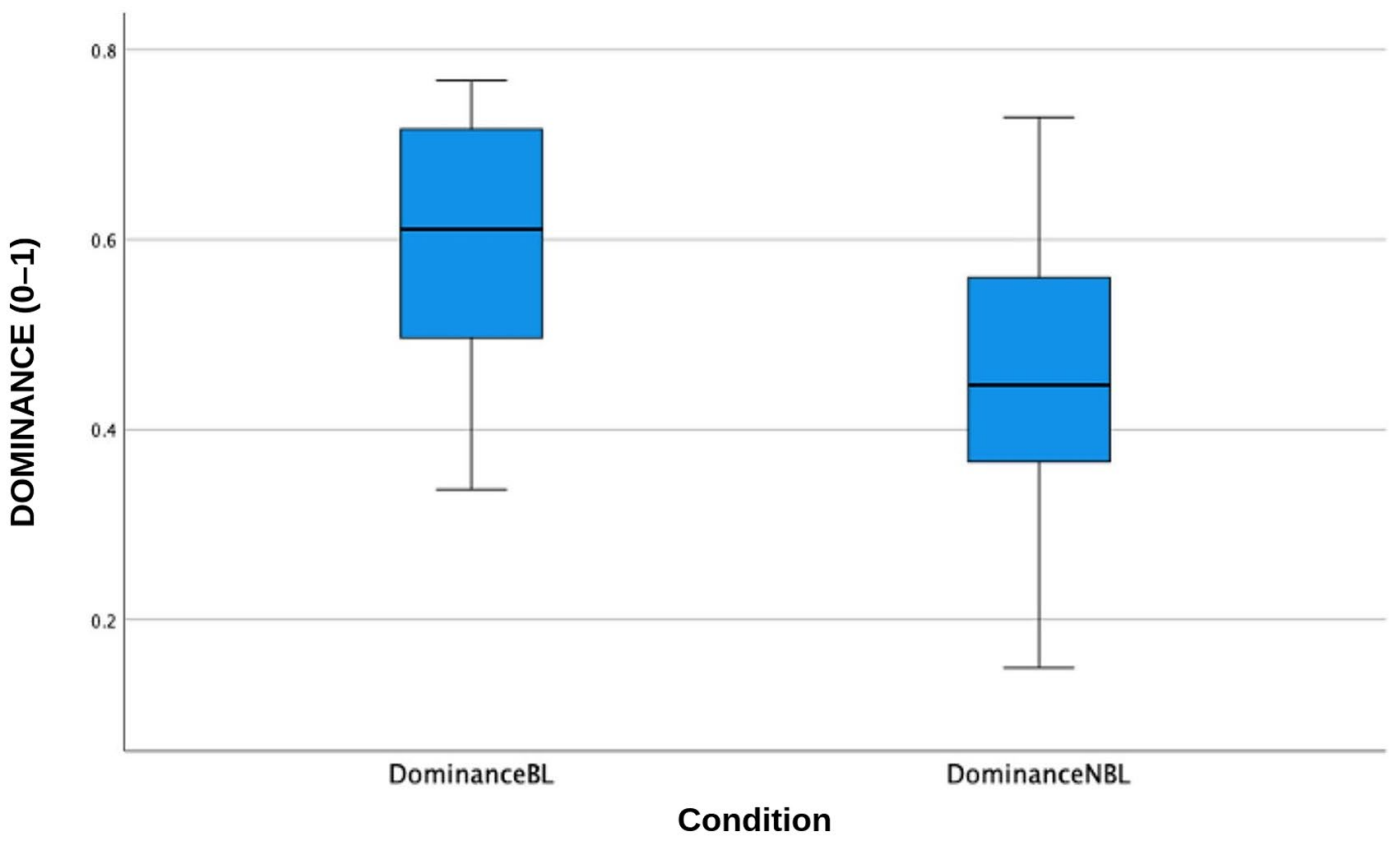
Dominance scores before (BL) and during (NBL) listening to “Howa Sahih” in the full sample. Dominance was significantly decreased during listening to the melody regardless of instrument and cultural background (*p* = 0.002).

Next, we were interested into investigating on whether listening to any of the three version of “Howa Sahih” played on Oud, Ney or Qanun, in the entire sample had any significant effect on valence, arousal or dominance. When not taking the cultural background into consideration, only dominance showed to be significantly affected by listening to the melody (NBL, referring to non-baseline, the music played on any of the three instruments) (Figure 2, Table 1, line 2).

Before and while listening to the music, EEG was recorded. Before listening to the music, the power of baseline frequency bands (theta, alpha, beta and gamma) was not significantly different between the two groups Arabs and non-Arabs (Table 1, line 12). When considering the entire population sample, listening to the music (NBL) was not associated to any significant change of theta or alpha frequency bands. However, listening to the melody “Howa Sahih” induced significant changes in beta and gamma frequency bands as compared to baseline frequency bands (Table 1, line 2).

**Figure 3:**
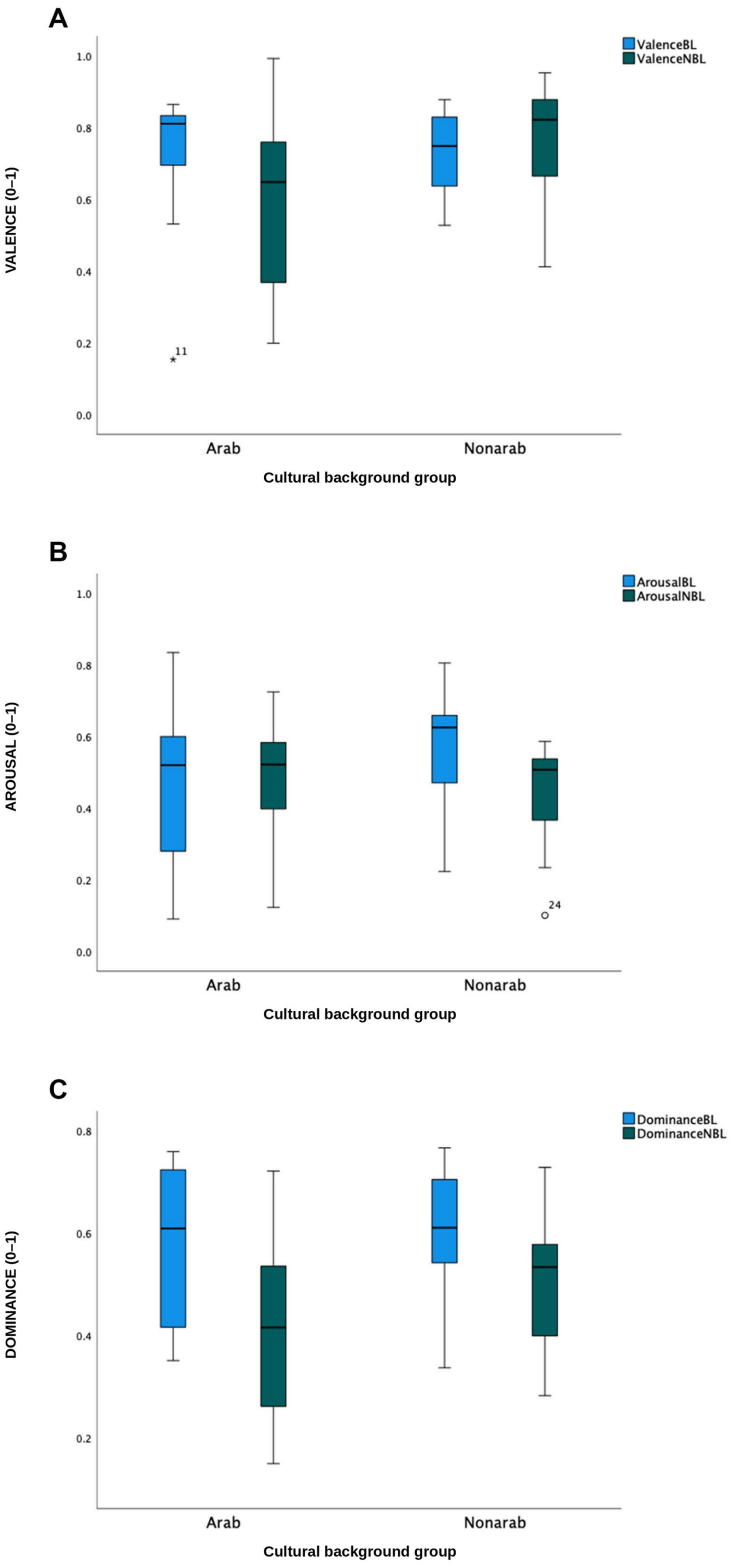
Emotion scores at baseline (BL) and during listening (NBL) to “Howa Sahih” in Arab and non-Arab groups. **(A)** Valence was significantly decreased in the Arab group (*p* = 0.030) but not in the non-Arab group. **(B)** Arousal was significantly decreased in the non-Arab group (*p* = 0.031) but not in the Arab group. **(C)** Dominance was significantly decreased in both groups (Arab: *p* = 0.039; non-Arab: *p* = 0.020).

However, when running statistical tests on the two groups Arabs and non-Arabs separately, listening to the melody “*Howa Sahih*” induced a significant decrease of both valence (Figure 3A) and dominance (Figure 3C) in individuals with Arab background, and a significant decrease of both arousal (Figure 3B) and dominance (Figure 3C) in individuals with non-Arab background. All *p*-values are listed in Table 1, line 7.

When it comes to frequency bands, while considering Arabs and non-Arabs separately, we observed that the power of beta bands while listening to music was significantly increased but only in the non-Arab group, while the significant increase of gamma bands was only observed in the Arab group (Table 1, line 7).

### Different instruments induced different EEG activity at T7 and emotions in response to the same melody

The same melody did not trigger the same effect when played on *Oud, Ney* or *Qanun*. When played on *Oud*, participants reported emotions with significantly decreased arousal (Table 1, line 8) and dominance scores in the non-Arab group as compared to their baseline (Table 1, line 8). However, no significant change to power of baseline frequency bands was observed in any of the groups (Table 1, line 8). Nevertheless, the power of gamma bands at T7 was still significantly increased in Arabs as compared to non-Arabs (Table 1, line 13).

When played on the *Ney*, the reported emotions had significantly decreased valence and dominance (Table 1, line 10) in the Arab group, and decreased arousal (Table 1, line 10) in the non-Arab group as compared to their respective baselines. A significant increase of gamma bands at T7 was observed in Arabs only and this difference was significant as compared to non-Arabs (Table 1, lines 10 & 15).

Finally, the melody on *Qanun* did not trigger any significant change to baseline neither in the Arab nor in the non-Arab group for any of the variables (Table 1, line 9). A significant increase of both beta and gamma bands was observed in the Arab group, but this difference was not significant as compared to the non-Arab group (Table 1, lines 9 & 14).

When testing the differences between Oud, Ney, and Qanun across all participants together, the Qanun elicited significantly greater arousal than either the Ney or the Oud (Table 1, lines 4–5).

### The melody played on Ney induced significantly different emotions and brainwaves between Arabs and non-Arabs

**Figure 4:**
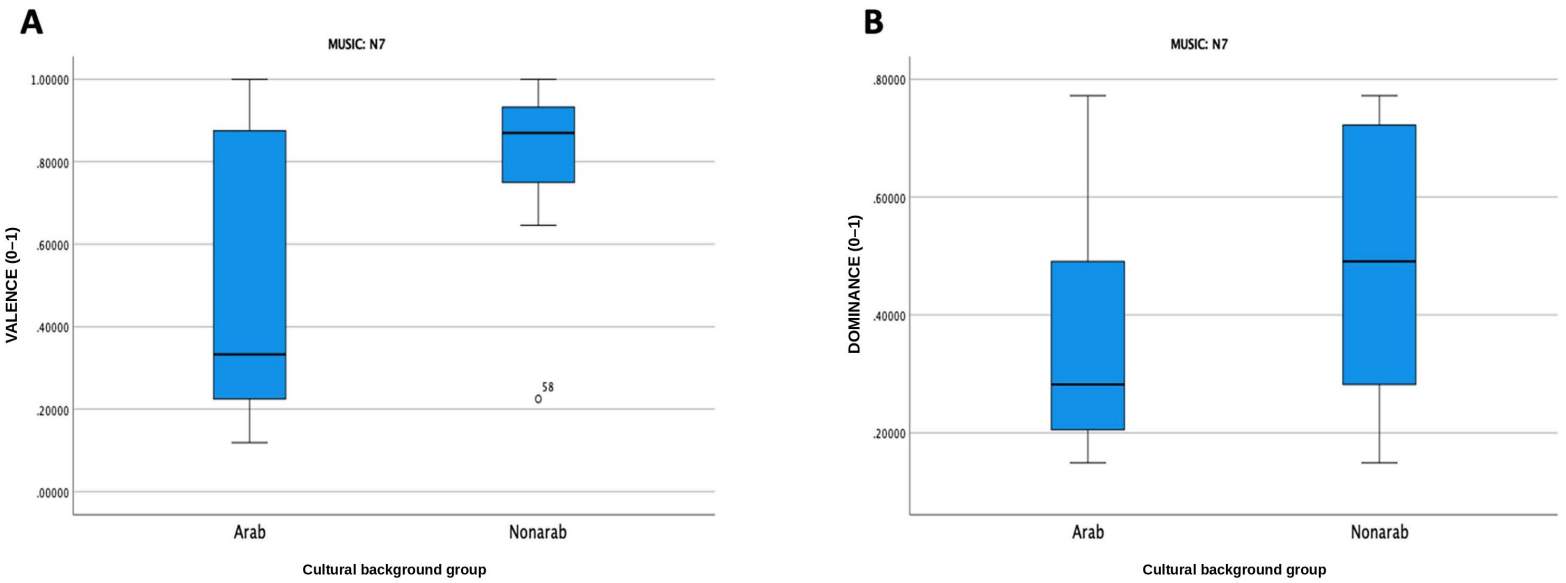
Emotion scores during “Howa Sahih” played on Ney (N7) in Arab and non-Arab groups. **(A)** Valence was significantly lower in Arabs than non-Arabs (*p* = 0.041). **(B)** Dominance was significantly lower in Arabs than non-Arabs (*p* = 0.049).

Arabs and non-Arabs reported significantly different emotions only when the melody was played on *Ney*. Indeed, when played on *Ney*, Arabs reported emotions with significantly lower valence (*p*-value = 0.041) and lower dominance (p-value = 0.049) than non-Arabs (Figure 4, Table 1, line 15).

However, when played on Oud or Qanun, participants did not report significantly different emotions between Arabs and non-Arabs. All *p*-values are listed in Table 1.

## Discussion

Understanding the effect of music on the human brain has always been a challenge because of all the variables that characterize a music and the complexity of the human brain. With advances in neurosciences, the growing field of neuromusicology is now gaining much interest as it allows a better understanding of the mechanisms underlying the use of music and sounds in many different fields.

Enculturation is the life-long process of learning in individuals in a specific community. Exposure to sounds during life will develop musical knowledge and memory of specific styles, forms, and structures available in the prevailing music. Encultured music is the music stored in our memories after a certain period of exposition to a certain culture. For example, the style of music listened to or sung by a mother in the presence of her child will be ingrained in the child’s memory as part of his/her culture. Culture not only affects the type of music listened to, but also includes the storing in memory of timbres of specific instruments, forms of music and pitch discrimination abilities through musical training and language. Therefore, listening to music may stir memories, together with its associated emotions. The choice of “Howa Sahih” was motivated by the intuitive expectation that individuals who are familiar with this song would probably feel differently than those who never heard it. Not only intuitive, since a recent study has indeed showed that familiarity matters (Bonomo et al., 2022). “Howa Sahih” is also a melody on the Saba Maqam and the main question was to find out whether individuals from different backgrounds would report emotions with significant different characteristics and EEG activity at T7. A summary of the results is displayed in Table 2.

**Table 2:** Summary of significant effects of listening to “Howa Sahih” on emotion scores and EEG frequency bands, by instrument and cultural background group.

Instrument used	In Arabs	In non-Arabs	In mixed population
Howa Sahih overall (regardless of instrument)	Decreases valence	Decreases arousal	Decreases dominance
	Decreases dominance	Decreases dominance	Increases beta bands
	Increases gamma bands	Increases beta bands	Increases gamma bands
Howa Sahih on Oud	NS	Decreases arousal	–
		Decreases dominance	
Howa Sahih on Ney	Decreases valence	Decreases arousal	–
	Decreases dominance		
	Increases gamma bands	Decreases dominance	
Howa Sahih on Qanun	Increases beta bands	NS	–
	Increases gamma bands		

NS, not significant.

The principal contribution of this work is methodological: it demonstrates that cultural background contributes to the emotional and electrophysiological responses to the same musical stimulus, and that failing to account for it may dilute effects in mixed samples—a possible explanation for the heterogeneity reported in music-intervention meta-analyses.

When considering all people, regardless of their cultural background and when considering the melody regardless of what music instrument it is played with, we observe that listening to 1 min of “Howa Sahih”: significantly decreased dominance, significantly increased beta bands, and significantly increased gamma bands. These results are consistent with the literature and provide further evidence that gamma bands are closely related to emotions (Gupta et al., 2023).

When comparing Arabs and non-Arabs, listening to the melody was shown to result in significantly decreased dominance in both Arabs and non-Arabs, significantly decreased valence in Arabs and significantly decreased arousal in non-Arabs as compared to their respective baselines, regardless of what music instrument it was played with.

Arabs and non-Arab backgrounds’ reactions to the music differed significantly for their: Valence on Ney, Dominance on Ney, Gamma bands on Ney and Gamma bands on Oud. We conclude that the Ney instrument is more likely to trigger emotional responses in Arabs than non-Arabs than both Oud and Qanun. Some explanation to the results might be that the Qanun and Oud might have close timbres to Harp and Guitar found in western music, both being string instruments.

These preliminary findings may inform future clinical research investigating whether culturally tailored music interventions using Maqam Saba may be relevant for conditions involving emotional dysregulation, including depression. A growing body of evidence suggests that sad music can produce beneficial emotional effects. Taruffi and Koelsch (2014) showed that listening to sad music leads to regulation of negative emotion and mood as well as consolation, and that the appreciation of sad music follows a mood-congruent fashion: individuals tend to choose music that matches their current emotional state. This mood-matching principle—known in music therapy as the ISO principle (Ratcliff et al., 2014)—is central to many therapeutic approaches, where patients are asked to select a piece of music that they perceive as matching their feeling. Sad music, in particular, appears to engage emotional processing rather than simply reinforcing sadness: Taruffi et al. (2017) demonstrated that sad music, compared with happy music, is associated with stronger self-referential cognitive processes and greater activation of the Default Mode Network, suggesting that sad music promotes introspection and internal emotional processing. Furthermore, Taruffi et al. (2021) showed that individuals with higher trait empathy exhibit stronger EEG responses to sad music, with recruitment of brain regions involved in compassion, mentalizing, and emotional regulation. Meta-analytic evidence further supports the efficacy of music-based interventions for depression and anxiety across clinical populations (Chou et al., 2026).

However, if sad music is to be used as a tool for emotional processing and regulation, then a fundamental question arises: what constitutes sad music for a given individual? The present study demonstrates that this question cannot be answered without considering the listener’s cultural background. The melody “Howa Sahih” on the Saba Maqam induced emotions with significantly decreased valence—consistent with sadness—only by Arab listeners, and only when played on the Ney. Non-Arab listeners did not report feeling the same sadness-associated response. This finding is of critical importance for music therapy: culture plays a central role in how music is experienced, and there is a clear need to develop therapeutic techniques that use tools adapted to specific populations.

The Maqam Saba is particularly well-suited for this purpose. Characterized by its distinctive interval signature—¾ tone, ¾ tone, ½ tone (Yaghmour et al., 2021)—the Saba Maqam contains a quartertone that is not typically present in Western music but is deeply embedded in the modal music traditions of the Middle East and North Africa. This quartertone gives the Saba Maqam a tonal quality that cannot be replicated using Western scales, and it may be precisely this feature that drives the culturally specific emotional response observed in our study.

Within the Arab group, timbre played a particularly important role. The Ney was the only instrument that produced significantly decreased valence and increased gamma activity as compared to baseline, and the only instrument for which the difference between Arab and non-Arab listeners was statistically significant for both self-reported emotions and EEG. The Oud and Qanun, while also traditional Middle Eastern instruments, did not produce the same pattern. This finding gives empirical support—and also an important nuance—to the long-held belief in Middle Eastern cultures that the Ney is inherently a sad instrument. Our results suggest that the Ney is not sad for everyone: it elicits sadness-associated responses specifically in individuals who are culturally familiar with its timbre and with the modal system in which it is played. The listener is therefore a central variable that must be considered in the composition of melodies intended for therapeutic use, and indeed for artistic expression as well.

Gamma oscillations, which were specifically increased in Arab listeners when the melody was played on the Ney, have also attracted considerable clinical interest beyond emotional processing. Gamma entrainment through sensory stimulation has been proposed as a novel non-pharmacological approach for Alzheimer’s disease (Manippa et al., 2022), and increased gamma activity has been associated with memory integration and higher cognitive processing (Gupta et al., 2023). The ability of the Saba Maqam played on Ney to reliably increase gamma power in Arab listeners warrants further investigation as a potential stimulus for gamma-based interventions in Arab populations.

Taken together, these findings provide preliminary evidence that developing culturally tailored music interventions—using Maqams, instruments, and melodies that are culturally meaningful to the target population—may be relevant to music-based interventions; this hypothesis will need to be tested through a logical progression of studies, from healthy samples to clinical populations involving conditions such as depression, grief, and post-traumatic stress, and, ultimately, controlled intervention trials.

### Limitations

Hearing music in the background passively is different than actively listening to it. For this reason, we suspect the increase in beta bands to be due to the fact that participants were asked to be mindful of their emotions, and that required thinking as they were listening.

The small sample size resulted in considerable variability within groups, as reflected by the overlapping interquartile ranges observed in some boxplots despite statistically significant between-group differences. Larger sample sizes in future studies would help confirm these findings and reduce within-group variability. Gamma bands have also been associated with higher brain functions such as cognition and memory. Therefore, at this stage of our analysis, we cannot determine whether the increased gamma bands observed in Arab listeners are directly associated with negative emotions or whether they constitute a confounding factor: Arab individuals might have recalled the memory of the song, which could have increased gamma power spectra independently of the emotional response. Furthermore, familiarity with the specific melody was not systematically recorded for all participants and could not be analyzed here; future studies should include a direct, systematic measure of prior familiarity with the stimulus. To address this, the remaining branch of the trial uses the same Maqam Saba presented as an improvisation—a melody unfamiliar to all listeners regardless of cultural background. A planned comparison between the present familiar melody and this unfamiliar improvisation will help disentangle whether the observed responses, in particular the increased gamma power in Arab listeners, are driven by the Maqam itself or by recognition of the familiar song, thereby addressing the familiarity–memory confound. Because recording was restricted to a single electrode (T7), the present findings are site-specific and cannot be generalized to whole-brain activity. Future studies should employ higher-density EEG, or methods with greater spatial resolution such as fMRI or fNIRS, to localize these responses.

Future studies could also employ neuroimaging methods with higher spatial resolution, such as fMRI, to clarify the neural basis of these responses and to test whether the gamma effect reflects emotional processing or memory retrieval. In addition, each stimulus in the present study lasted 1 min; future work should vary stimulus duration to characterize possible dose–response dynamics—that is, whether responses amplify, plateau, or habituate with longer exposure.

Spectral power was expressed in decibels (dB) rather than in absolute units. Values in the higher-frequency bands—particularly gamma—clustered close to the lower bound of the recording scale (≈ − 80 dB), indicating a floor effect in which gamma amplitude approached the resolution limit of our acquisition configuration. Two features of the setup likely contributed: the sampling rate of 128 Hz, with an acquisition bandwidth of 0.16–43 Hz, placed the upper part of the analyzed gamma range (25–45 Hz) at the edge of the recorded band; and the two-channel scalp montage offers a limited signal-to-noise ratio for the intrinsically low-amplitude gamma signal. Consequently, although between-group differences in gamma reached statistical significance on rank-based tests, the absolute gamma differences were small and should be interpreted with caution, as exploratory pilot-level findings rather than precise estimates of gamma power. Future studies should use a higher sampling rate (≥256–512 Hz) with a bandwidth extending well above the gamma range, higher-density EEG to improve signal-to-noise ratio, and spectral power expressed in absolute (μV^2^) or baseline-normalized units rather than on a bounded dB scale, to avoid this floor effect.

No compensation was offered for participation. While unpaid volunteering may influence sample composition, our protocol required participants to remain still during EEG recording and to introspect sincerely on their emotional state—a subjective task whose data quality depends on genuine engagement. In this context, non-compensated enrolment helped ensure that participants took part out of authentic interest rather than financial incentive, reducing the risk of inattentive or rushed responding. We acknowledge, nonetheless, that any volunteer sample limits generalizability, and future studies with more constrained or objective tasks might adopt compensated recruitment.

Additionally, the categorization of participants into Arab and non-Arab groups was based on self-reported ethnicity collected during the enrollment process rather than through a standardized ethnicity questionnaire. While the grouping reflects meaningful cultural distinctions in terms of musical exposure and enculturation, future studies should include a validated instrument for assessing cultural and ethnic background, as well as direct questions on prior familiarity with the specific melody and instruments used. The binary Arab/non-Arab grouping does not capture regional diversity; future studies with much larger stratified sample, could test whether the effect reflects specific repertoire familiarity rather than a broad cultural category.

Probably, the most important limitation that readers must take into account is the following: participants were asked to “select the emotion that best describes their feeling after listening to the audio presented.” Researchers have distinguished the perceived from the felt emotion, a quite tricky distinction that indicates that a music might sound sad and still result in a pleasant feeling. When someone listens to music and tells us about their feeling, it is hard to find out whether the emotion they are reporting is about how they feel or what they think about the music. We expect that, despite the instruction and given the short time they had to provide a response, some participants might have selected the emotion that they first perceived as they listened to the music, which would be more about what emotion they associated to the music rather than how they actually felt. This will remain an unanswered question. However, the EEG data, which are more objective, agree to say that gamma bands are increased at T7 for Arab listeners, particularly when the melody was played on the Ney, which supports observations in the case study of a performer playing the Maqam Saba (Yaghmour et al., 2021).

Finally, timbre, music forms, pitch discrimination, encultured music are all important cultural-related factors that can influence perception of the music listened to, as well as our representation of it. The actual emotions that are triggered by a specific music therefore depend on the memory, cognitive processes and perception of music. However, the labelling of the emotion is strongly dependent on language and representations derived from the music and is therefore also dependent on culture. This leads to important consequences on how studies that do not take culture into consideration are being interpreted. In the present study specifically, emotions were mapped using the NRC VAD Lexicon, an English-language resource, which may not fully capture culturally specific connotations in a linguistically diverse sample where no single native-language instrument could apply to all participants.

Results indicate that future studies should consider the cultural background parameter as a fundamental variable before engaging in statistical analyses. Clinical studies using music often yield conflicting results, and this might be one possible explanation: diversifying ethnicities in the sample population might actually dilute any possible significant outcome, and result in non-significant results when in fact important observations could have been made with a tailored study design.

## Data Availability

The raw data supporting the conclusions of this article will be made available by the authors, without undue reservation.

## Supplementary material

Supplementary Audio 1 Recording of "Howa Sahih El Hawa Ghalab" played on Oud (O7) by Abdalla Mhoul (Qatar Music Academy).

Supplementary Audio 2 Recording of "Howa Sahih El Hawa Ghalab" played on Ney (N7) played by Yassine Ayari (Qatar Music Academy).

Supplementary Audio 3 Recording of "Howa Sahih El Hawa Ghalab" played on Qanun (Q7) played by Taoufik Mirkhan (Qatar Music Academy).
